# Efficacy of dapagliflozin in the treatment of HFrEF with obstructive sleep apnea syndrome (DAHOS study): study protocol for a multicentric, prospective, randomized controlled clinical trial

**DOI:** 10.1186/s13063-023-07332-x

**Published:** 2023-05-09

**Authors:** Liang Xie, Songsong Song, Shengnan Li, Qin Wei, Hong Liu, Chao Zhao, Fuchao Yu, Jiayi Tong

**Affiliations:** 1 School of Medicine, Southeast University, Nanjing, China; 2 Department of Cardiology, Jinling Hospitial, Affiliated Hospital of Medical School, Nanjing University, Nanjing, China; 3 Department of Cardiology, Zhongda Hospital, Nanjing, China; 4 State Key Laboratory of Bioelectronics, School of Biological Science and Medical Engineering, Southeast University, Nanjing, China

**Keywords:** Heart failure, Dapagliflozin, Sleep apnea, Sleep-disordered breathing

## Abstract

**Background:**

Heart failure with reduced ejection fraction (HFrEF) is associated with sleep dyspnea (SDB), which plays an adverse role in the pathophysiology of the condition. SDB management in HFrEF, however, remains controversial. HFrEF’s medical management has recently made significant progress with the discovery of new therapeutic avenues, namely sodia-glucose cotransporter-2 (SGLT-2) inhibitors, and better treatment of co-morbidities. Dapagliflozin, one of the SGLT-2 inhibitors, is a good candidate for correcting SDB of HFrEF patients because their known mechanisms of action are likely to counteract the pathophysiology of SDB in HFrEF.

**Methods/design:**

The trial is a 3-month, multicentric, prospective, randomized controlled clinical study. Patients (i.e., adults with left ventricular ejection fraction ≤ 40%, Apnoea–Hypopnoea Index ≥ 15) will be randomized to receive optimized heart failure therapy plus a standard dose of dapagliflozin, while the control group will receive only optimized heart failure therapy. Patients will be evaluated before and after 3 months (nocturnal ventilatory polygraphy, echocardiography, laboratory testing, and quality-of-life and SDB questionnaires). The primary outcome is the change in the Apnoea–Hypopnoea Index, before and after 3 months of treatment.

**Trial registration:**

www.chictr.org.cn, ChiCTR2100049834. Registered 10 August 2021.

## Background

Heart failure (HF) refers to the end stage of various structural or functional diseases of the heart [1]. An estimated 64.3 million people are living with heart failure worldwide [2]. In developed countries, the prevalence of known heart failure is generally estimated at 1 to 2% of the general adult population [3]. A high proportion of patients with heart failure combined with sleep dyspnea accounts for 50–75% of the total number of patients with heart failure [4, 5]. Sleep apnea is a disease characterized by interrupted breathing at night, easy arousal, daytime sleepiness, and reduced blood oxygen saturation. It is mainly divided into central sleep apnea syndrome (CSAS) and obstructive sleep apnea syndrome (OSAS) [6, 7]. Patients with sleep disordered breathing (SDB) suffer from repeated hypoxia due to interrupted or weakened breathing during sleep, which causes sympathetic nerve excitation and impairs the function of vascular endothelium, thus affecting the cardiovascular system of the patients. Patients with heart failure combined with sleep apnea who did not receive further treatment had twice the risk of death as those without SDB, which was mainly associated with an increased incidence of malignant ventricular arrhythmias [8]. In patients with concomitant ischemic cardiomyopathy, untreated consequences are more severe, with a threefold increase in mortality [9, 10]. Currently, obstructive sleep apnea has been identified as a risk factor for heart failure and exacerbation.

At present, the treatment of chronic heart failure has made a milestone progress. Despite this, 1 ~ 2% of adults in the world still suffer from heart failure, with a high incidence rate, and the resulting hospitalization rate, fatality rate, and social and economic burden also increase year by year [11]. Dapagliflozin, a SGLT-2 inhibitor, was found to have a unique role as a hypoglycemic agent in the treatment of heart failure, especially in patients with reduced ejection fraction. DAPA⁃HF was a multicenter, prospective randomized controlled trial involving 4744 patients with chronic heart failure (LVEF < 40%) from 20 countries, of whom 2605 (54.9%) were non-diabetic, 1097 (23.1%) were Asian, and 237 (5.0%) were from China. Results showed that dapagliflozin was associated with a significant reduction in the primary composite end point compared with placebo [12]. Dapagliflozin is clearly indicated in the domestic label for use in adult patients with heart failure (HFrEF) in patients with reduced ejection fraction (NYHA II-IV) to reduce the risk of cardiovascular death and hospitalization due to heart failure [13, 14].

Dapagliflozin has also been shown to be effective in patients with sleep apnea and diabetes. From the mechanism, it is not difficult to guess that dapagliflozin may have a potential effect of improving the ventilation of OSAHS patients. In recent years, it has been partially verified in some studies. For type 2 diabetes patients complicated with obstructive sleep apnea (OSA), dapagliflozin not only improves blood glucose, blood pressure, blood lipids, and weight loss but also improves ventilation function better than glimepiride [15]. In Yi Tang’s case control study [16], 36 newly diagnosed patients with T2 diabetes who also had OSA were randomly assigned to two groups. The effects of dapagliflozin can be significant in decreasing blood glucose, body mass index, blood pressure, and blood pressure AHI, improving hypoxemia during sleep, and reducing excessive daytime sleepiness, so it has the potential to be an effective treatment for OSA. A prospective, open-label, single-arm, multicenter trial showed improvement in moderate to severe SDB as well as mild SDB [17].

Few studies have reported the pharmacological treatment of sleep apnea especially with heart failure [18–20]. We hypothesized that dapagliflozin, as a new star in the treatment of heart failure, could improve sleep apnea in heart failure patients. In our study, patients with heart failure complicated with moderate to severe OSA (AHI equal or greater than 15) [21] and reduced ejection fraction were enrolled, and dapagliflozin was added to the conventional optimized heart failure treatment group. This is to observe and evaluate the changes of OSAH-related indicators and the treatment of heart failure and verify the role of dapagliflozin in the treatment of heart failure complicated with OSA.

## Methods/design

### Study design

This study is a prospective, multicenter, randomized controlled superiority trial. The study will be conducted in two research centers (Zhongda Hospital and Jinling Hospital). This paper presents the design and protocol for the trial according to the Standard Protocol Items: Recommendations for Interventional Trials (SPIRIT) statement [22].

**Fig. 1:**
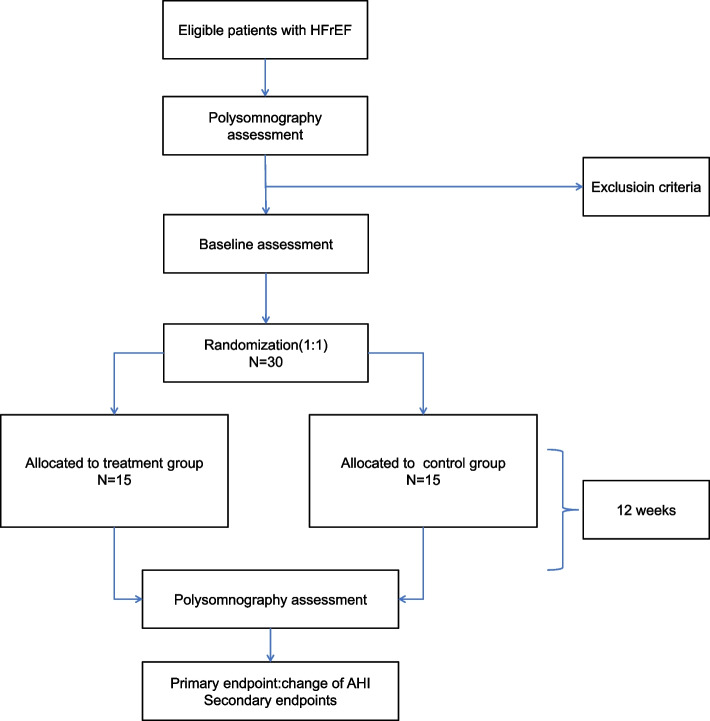
Study design overview

An overview of the study design and timeline for participants is provided in Fig. 1. Those eligible for dapagliflozin treatment and treated in real-life conditions will be invited to participate. Following the inclusion and exclusion criteria, a pre-therapeutic evaluation will be conducted [physical examination, echocardiography, laboratory testing, Minnesota Living with Heart Failure Questionnaire and EuroQol Group 5-Dimension Self-Report Questionnaire (EQ-5D), Epworth Sleepiness Scale, nocturnal ventilatory polygraphy] [20].

Patients will be divided into two groups:

- Group 1: patients received optimized heart failure therapy plus dagaglizin 10 mg daily, dapagliflozin will be started the day after the polygraphy
- Group 2 (control group): patients received only optimized heart failure therapy

“Optimal drug therapy” in large heart failure trials usually refers to a combination of BBL, ACE inhibitors or ARBs and MR antagonists, with an optimal dose [23]. After 3 months of treatment, the final evaluation will include physical examination, echocardiography, laboratory testing, Minnesota Living with Heart Failure Questionnaire and EQ-5D, Epworth Sleepiness Scale, and nocturnal ventilatory polygraphy for all patients included in the study. During the research process, the participants cannot take other types of SGLT2 inhibitors such as empagliflozin and canagliflozin. Using ventilators to treat sleep apnea is not allowed; if it is necessary, they will withdraw from the study.

### Eligible participants

The outpatient or inpatient cases of cardiology department of Zhongda Hospital and Jinling Hospital were selected. Patients to be enrolled must meet all inclusion criteria listed in Table 1 and not meet any of the exclusion criteria. The inclusion criteria and exclusion criteria are listed in Table 1. A list of patients who recently underwent echocardiography and polysomnography at the department of cardiology and signed a form consenting to participate in research will be generated. Those patients with an apnea–hypopnea index greater than 15 events/hour and left ventricular ejection fraction less than 40 will be selected and contacted by telephone. The acceptors will be asked to visit the research center where the informed consent process will be conducted.

**Table 1:** Inclusion and exclusion criteria

Inclusion criteria	Exclusion criteria
1) Voluntarily participate, understand and sign the informed consent;	1) Have participated in other clinical studies
2) Symptomatic chronic heart failure, NYHA grade II–IV, chronic heart failure diagnosis according to ESC Guidelines for Heart Failure 2016 and China Guidelines for Heart Failure 2018; the condition is relatively stable, without intravenous medication	2) Hemodynamic instability
3) LVEF 40% or less	3) Allergic to drugs or their ingredients
4) Over 18 years old, less than 80 years old	4) Acute myocardial infarction
5) The patient has received standard optimized treatment for HFrEF and is treated according to the recommended drug dose in the guidelines, unless contraindication or intolerance	5) Active liver disease or severe liver insufficiency
6) eGFR ≥ 45 ml/min/1.73 m^2^	6) Known active infection or severe blood disease or endocrine dysfunction
7) Diagnosis of OSAHS according to the Diagnostic Guidelines for Adult Obstructive sleep apnea (2016); AHI ≥ 15 events/h	7) History of COPD, asthma, or other serious lung diseases
	8) History of stroke, peripheral or central nerve abnormalities, cerebrovascular disease
	9) Pregnant and lactating women
	10) HbA1c ≥ 7.0%
	11) Previous use of dapagliflozin

Screening for GFR estimates < 30 mL/min/1.73 m^2^ as measured by a simplified MDRD formula using serum creatinine level, age, sex, and race [24]

### Follow-up and duration of the study

There will be two visits at the clinical research center, one before (baseline) and one after (3 months follow-up) the dapagliflozin intervention. Data collection to assess efficacy of the intervention will be managed by research team members. An overview of the measures used at each of the four time points is illustrated in Table 2. For improving the compliableness of patients, the strategies are as follows: (1) adhere to the principle of voluntary participation and explain the benefits of this study to the participants and the importance of treatment and adherence to the treatment. Health education process could increase the related knowledge, change the behavior, and enhance the compliance of patient. (2) Written informed consent will be obtained from each patient before they begin the trial. All participants will fill in informed consents. (3) The treatment is free for participants, such as the relevant examination expenses and the telephone bill, traffic expenditures, and analgesic drugs incurred during the treatment and follow-up. (4) Record participants’ contact methods in detail, including mobile phone, WeChat, and e-mail for contact and follow-up.

**Table 2:** Administration of study measures by assessment period

Research phase	Screening and randomization	Treatment period		
Interview	1	2	3	4
Research date	− 7–0	28 ± 2	56 ± 4	84 ± 6
Baseline information				
Informed consent	●			
General information	●			
History	●			
Physical examination	●	●	●	●
Treatment				
Inclusion/exclusion criteria	●			
Combined medication	●	●	●	●
Compliance		●	●	●
Completion of research				●
Inclusion/exclusion criteria				
PSG	●			
Echocardiography	●			
Security index				
Blood routine	●			●
Urine routine	●			●
Blood biochemical	●			●
Electrocardiogram	●			●
Adverse event assessment		●	●	●
Outcome				
AHI	●			●
Epworth Somnolence Scale (ESS)	●	●	●	●
Echocardiography	●			●
NT-proBNP	●			●
Quality of life as measured by the Minnesota Living with Heart Failure Questionnaire	●	●	●	●
EQ—5D—3L				
L_SpO2_	●			●
Inflammatory factors	●			●

### Sample size

The sample size calculation for this trial was performed using the PASS 11.0 program [25]. The primary end point of the main study was the decreasing value of Apnoea–Hypopnoea Index (△AHI). Based on existing data, a 8 ± 6 events/h change in AHI events/h between the control and therapy groups was considered clinically relevant (assuming that AHI would not change in the control group) [26–28]. Based on a power of 90% and an alpha value of 5%, it was calculated that a sample size of 30 patients (considering the 20% sample loss rate) would be sufficient to detect a difference in the effect size of at least 10%. Data monitors from the two centers will supervise the study process at a fixed period.

### Randomization process

As the sample size of the study was not large, we adopted a simple random method. Participants will be assigned a randomization code according to their sequential numbers and randomly allocated into one of two groups with a 1:1 ratio in the operating room.

### Study drug

Patients in the experimental group received a standard dose (10 mg daily) of dapagliflozin in addition to the standard and optimized heart failure regimen, while patients in the control group received the standard and optimized heart failure regimen alone. Subjects will be advised to take the study drug at the same time everyday according to approved instructions.

### Outcomes

#### Primary outcome

The primary endpoint of the trial is the AHI change determined by nocturnal polygraphy, before and after 3 months of treatment with dapagliflozin. The 2012 American Academy of Sleep Medicine recommendations are used to characterize apnea and hypopnea events, the phenotype, whether central, obstructive, or mixed.

#### Secondary outcomes

1. The proportion of patients with a 20% decrease in their AHI, before and after dapagliflozin
2. The proportion of patients with a 50% decrease in their AHI, before and after dapagliflozin
3. Epworth Somnolence Scale (ESS) score before and after treatment
4. Echocardiographic measures of structure and function before and after dapagliflozin [ejection fractions, left ventricular diameters, atrial surface, diastolic function (strain), and filling pressures]
5. Serum BNP and pro-BNP concentrations before and after dapagliflozin
6. Laboratory testing also included creatinine, potassium, sodium, hemoglobin, and alanine and aspartate transaminase
7. Quality of life as measured by the Minnesota Living with Heart Failure Questionnaire and EQ-5D-3L Questionnaire, before and after dapagliflozin
8. Levels of inflammatory and oxidative stress factors IL-6, CRP before and after dapagliflozin

### Adverse events

The adverse events will be investigated by open questions, including general symptoms, and by specific questions regarding symptoms potentially related with the drugs used in the trial, as hypotension, frequent urination, urgent urination, fatigue, loss of appetite, nausea, vomiting, etc. The rate of adverse effects will be determined by comparing the frequency of adverse events in the experimental group and control group. The criteria for discontinuing the trial for an individual participant are as follows:

1. Those who have serious adverse reactions in the study and are not suitable to continue to participate in the study
2. During the study period, the participants had other acute and severe diseases and needed to take emergency measures

(The reasons and time of withdrawal from the study will be recorded in detail. Participants who have exceeded 1/2 of the course of treatment will be included in the curative effect analysis.)

Researchers will do their best to prevent possible dangers caused by this study. If adverse events occur, the medical expert committee will identify whether they are related to drug treatment or research process. The project will provide treatment expenses and corresponding economic compensation for the damage related to the trial in accordance with the Criterions for the Quality Control of Clinical Trial of Drugs.

### Data entry and management of data files

All data are entered into computerized database with the SAS software, V.9.3 (SAS Institute, Cary, North Carolina, USA). Values that are out of range or represent errors of faulty logic are avoided by double check. All participants’ data and patient health information will be confidential and protected during and after the trial. A code will be used to identify study participants, which will not be given to anyone outside of the study staff unless required by law. All paper records are stored in an area with two locked doors and/or drawers. Only the study staff will have access to the locks. All computerized records are password protected available only to the study team. We will use a data monitoring committee to provide oversight on integrity of the collected data.

### Ethics approval

All participants will be asked to sign the approved informed consent form prior to participation in the study. The study protocol (protocol version 2.0, issue date: 5 July 2021) was approved by the Ethics Committee of the Zhongda Hospital and Jinling Hospital under the number 2021ZDSYLL169-P01. The trial is conducted in keeping with Good Clinical Practice Guidelines, the principles outlined in the Declaration of Helsinki, and applicable local laws and regulations. The trial is registered at www.chictr.org.cn under the identification number ChiCTR2100049834. In the study, significant protocol modifications will be completed by the project applicants and primary investigators and resubmitted to the Ethics Committee for final review. After revising the program, there are researcher trains. If participants are involved, we will re-sign informed consent. We will perform audits to ensure that study protocol is followed every three months. Project management group meeting will be conducted both weekly and monthly. Weekly meeting will be held every Friday. Monthly meeting will be conducted at the last day of every month. The project management group will audit information on the research. The ethics committee will meet every 6 months.

### Dissemination

The final clinical report will be the basis for the study to be published in a medical journal and presented at national or international conferences. A person chosen by the lead investigators will jointly publish a formal report or publication of the study’s data. A report of the results of this study will be sent to the guardians of participants by mail. To ensure the scientific dissemination and transparency, de-identified data required to replicate our study can be supplied upon request with data usage agreements.

### Statistical methods

Outcomes will be analyzed by intention-to-treat and per-protocol. A low attrition rate is expected, since the interventions are of short duration and minimal risk. Missing data will be considered as missing. Data imputation will not be employed. Normally distributed data will be presented as means and standard deviation. Non-normally distributed data will be displayed as median and interquartile range. The significance of the differences between two groups will be tested by chi-squared test for categorical variables and t test for measurement data. Results with a *P* value lower than 0.05 for alpha error will be considered statistically significant. Statistical analyses will be performed using the SPSS 26.0 software (SPSS Inc., Chicago, IL, USA). We will do an interim analysis when the number of patients reaches half that.

## Discussion

As a result of untreated OSA, hypertension, cardiovascular disease, metabolic disorders, and neuropsychiatric problems can be significantly worsened [29]. As a result of periodic airway collapses, OSA causes sleep fragmentation and intermittent hypoxia. These phenomena lead to an increase in oxidative stress, reactive oxygen species formation, increased inflammation via the NF-kB pathway [30], sympathetic activation [31], metabolic dysregulation, and endothelial dysfunction [32] resulting in insulin resistance [33], hypertension, and cardiovascular diseases.

In patients with symptomatic OSA, positive airway pressure (PAP) is the primary treatment option. Treatment of OSA with PAP has some limitations. Patient adherence is reported to be poor with an averagemask use of 4.4 h/night [34]. Additionally, CPAP therapy has a limited effect on blood sugar control. There are currently no pharmacological agents listed in the treatment protocols for OSA, and medication use is restricted to the prevention and correction of precipitating conditions such as obesity and hypothyroidism [35, 36]. There is growing evidence that SGLT-2 inhibitors may reduce OSA severity [16, 17, 37, 38]. SGLT2 inhibitors may benefit patients with OSA by promoting weight loss. OSA severity can be improved by reducing weight and reducing diet alone. This effect is facilitated by SGLT2 inhibitors by increasing lipolysis and decreasing central obesity [38, 39]. As fat deposits around the neck and thorax may worsen upper airway collapsibility, weight loss may have a beneficial effect on airway collapse and AHI. However, the meta-analysis by Mir et al. included 65 patients with OSA and T2DM, and showed that AHI, BMI, and systolic blood pressure (SBP) all decreased and SpO_2_ increased with SGLT2 inhibition, though these results were not statistically significant [40].

Little is known about the effect of dapagliflozin on sleep apnea in patients with heart failure. None of the studies found in an ample PubMed and Embase search mentioned any randomized controlled trials (RCTs) of treatments for dapagliflozin is recognized as the most effective intervention to improve the severity of sleep apnea in patients with heart failure. DAHOS study is the first study designed to determine dapagliflozin’s effects on sleep apnea in patients with heart failure and provides an important basis for pharmacologic treatment of sleep apnea. Our hypothesis is that dapagliflozin significantly improved the severity of sleep apnea in patients with heart failure.

A limitation of this study is the sample size is not large enough, and the follow-up time is 3 months. Another limitation is that the study did not use double blindness and placebo.

In summary, our RCT will provide data for analyses of the relationship between OSA and dapagliflozin, which may be a potential treatment for sleep apnea.

## Trial status

At the time of manuscript submission, the enrollment of volunteers is ongoing. The study has been ongoing from October 2021. The protocol version is 2.0 (issue date 5 July 2021).

## Data Availability

Data and materials can be obtained from the corresponding author after the trial.
